# Roscovitine-induced Apoptosis in Neutrophils and Neutrophil Progenitors Is Regulated by the Bcl-2-Family Members Bim, Puma, Noxa and Mcl-1

**DOI:** 10.1371/journal.pone.0079352

**Published:** 2013-11-01

**Authors:** Sanjivan Gautam, Susanne Kirschnek, Michael Wiesmeier, Juliane Vier, Georg Häcker

**Affiliations:** Institute for Medical Microbiology and Hygiene, University Medical Center, Freiburg, Freiburg, Germany; University of Tübingen, Germany

## Abstract

Neutrophil granulocyte (neutrophil) apoptosis plays a key role in determining inflammation in infectious and non-infectious settings. Recent work has shown that inhibitors of cyclin-dependent kinases (cdk) such as roscovitine can potently induce neutrophil apoptosis and reduce inflammation. Using a conditional Hoxb8-expression system we tested the participation of Bcl-2-family proteins to roscovitine-induced apoptosis in mouse neutrophils and in neutrophil progenitor cells. Bcl-2 strongly protected against roscovitine-induced apoptosis in neutrophils. The isolated loss of either Bim or noxa provided significant, partial protection while protection through combined loss of Bim and noxa or Bim and Puma was only slightly greater than this individual loss. The only substantial change in protein levels observed was the loss of Mcl-1, which was not transcriptional and was inhibited by proteasome blockade. In progenitor cells there was no protection by the loss of Bim alone but substantial protection by the loss of both Bim and Puma; surprisingly, strongest protection was seen by the isolated loss of noxa. The pattern of protein expression and Mcl-1-regulation in progenitor cells was very similar to the one observed in differentiated neutrophils. In addition, roscovitine strongly inhibited proliferation in progenitor cells, associated with an accumulation of cells in G2/M-phase.

## Introduction

Neutrophil granulocytes (neutrophils) are produced at a high rate in the bone marrow (approximately 10^11^ per day in healthy humans) and released into the peripheral blood [1]. This massive production is counter-acted by rapid apoptosis although the precise life-span of neutrophils in human peripheral blood is contentious at present [2-4]. Apoptotic neutrophils are cleared by macrophage-mediated phagocytosis, which has anti-inflammatory effects and may contribute to the resolution of inflammation [5]. On the other hand, apoptosis appears to be the only mechanism that physiologically terminates neutrophil activity. If apoptosis is experimentally inhibited, neutrophils continue to function and in the presence of microbial stimuli maintain their pro-inflammatory activity [6]. Importantly, numerous microbial and cellular, host-derived inflammatory mediators can inhibit apoptosis in neutrophils, which very likely prolongs their activity at inflammatory sites [1].

Modulation of neutrophil apoptosis is therefore an attractive way to modulate inflammation, and experimental data in animal models support the validity of such an approach. Infusion of apoptotic neutrophils has been found to have a strong anti-inflammatory effect in mice [7]. Neutrophils can be driven to undergo apoptosis by the TNF-family member TRAIL, and recombinant TRAIL has been shown to be able to reduce neutrophil numbers and inflammation in mice *in vivo* [8]. An intriguing approach is the application of drugs that were developed to inhibit cyclin dependent kinases (CDKs). These compounds were developed as anti-cancer drugs and have been shown to have multiple biological effects in various cellular models, such as inhibition of transcription, activation of p53 and inhibition of NF-κB [9]. A number of CDK-inhibitors have been shown to induce apoptosis very efficiently in neutrophils [10]. R-roscovitine [here referred to as roscovitine] is the substance of this group that has been investigated in consecutive studies. 

Roscovitine has been shown to have remarkable activity in the reduction of inflammation in animal models of sterile inflammation [10]. In pneumococcal meningitis, when given together with antibiotics, roscovitine could reduce neutrophil-numbers and neutrophil-mediated tissue damage [6], and lung inflammation induced by pneumococci or by lipoteichoic acid could also be ameliorated by the application of roscovitine [11].

The mechanism of roscovitine-induced apoptosis has been the subject of several studies. Human neutrophils isolated from peripheral blood have been found to express CDK1, 2 and 5 [10] although a later report concluded that targeting of CDK7 and 9 by roscovitine is more relevant to roscovitine-induced apoptosis [12]. Roscovitine-induced apoptosis in mouse neutrophils is blocked by Bcl-2 [6], which demonstrates that the mitochondrial apoptosis pathway is used. In tumour cells, roscovitine induces apoptosis that is accompanied by down-regulation of the anti-apoptotic Bcl-2-family member Mcl-1 [13,14], and roscovitine also down-regulates Mcl-1 in neutrophils [10,12,15]. Since Mcl-1 is critical for neutrophil survival [16], this is probably a relevant pro-apoptotic mechanism of roscovitine-action. 

The down-regulation of both Mcl-1-mRNA and -protein by roscovitine-treatment of neutrophils has been demonstrated in human neutrophils [10,12]. However, co-treatment with the proteasome-inhibitor MG-132 appeared to maintain the protein levels of Mcl-1 completely in the presence of roscovitine, which may be taken to suggest that Mcl-1 mRNA-regulation is not a critical effect of roscovitine [12]. The regulation of Mcl-1 protein levels may occur on various levels, including transcriptional, translational and post-translational mechanisms [17]. The Mcl-1-stabilising effect of proteasome inhibition indicates a post-translational effect of roscovitine-treatment. 

Mcl-1 is a member of the Bcl-2-family of proteins, where it (like Bcl-2) has an anti-apoptotic function. The Bcl-2-family of proteins collectively regulate mitochondrial apoptosis. Mitochondrial apoptosis occurs when the concerted action of the Bcl-2-protein family leads to the permeabilisation of the outer mitochondrial membrane, permitting the release of cytochrome *c* from the intermembrane space into the cytosol, where it binds Apaf-1, initiating caspase-activation. The Bcl-2-family consists of three groups of proteins, one anti- and two pro-apoptotic [18]. The initiators are the pro-apoptotic BH3-only proteins (8 proteins are typically considered BH3-only proteins, including Bim, Puma and Noxa). BH3-only proteins activate the effector proteins Bax and/or Bak. Bax and Bak can in many situations replace each other; the molecular details of their activation by BH3-only proteins is still not entirely clear but involves the direct activation of Bax by some BH3-only proteins (Bim, Puma, Bid) [19,20] and very likely the release of Bax/Bak from inhibition by anti-apoptotic Bcl-2-proteins [21]. The inhibitory function of anti-apoptotic Bcl-2-proteins (Bcl-2, Bcl-XL, Bcl-w, Mcl-1, A1) is exerted by direct binding and inhibition of probably mostly BH3-only proteins but also Bax and Bak [22].

Neutrophils are relatively difficult to investigate. Human neutrophils can be easily isolated from peripheral blood but may be at varying stages of their life and cannot be genetically modified efficiently. Mouse neutrophils can be isolated but are relatively few in number and die quickly in culture. Drugs like roscovitine likely also have effects on neutrophil progenitors in the bone marrow but these progenitors cannot be isolated in a pure form in large numbers.

An experimental model for the study of neutrophils has been introduced recently that uses the expansion of committed mouse neutrophil progenitor cells *in vitro*. In this model, conditionally active (regulated by its fusion to the oestrogen-receptor ligand-binding domain) Hoxb8 permits unlimited growth of these cells, which upon turning off Hoxb8 by oestrogen-withdrawal differentiate over about 4 days to mature neutrophils *in vitro* [23]. Cells can be established from genetically modified mice, and the model permits the study of cells almost indistinguishable from mature neutrophils. It also allows the investigation of neutrophil progenitor cells from a stage onwards that by marker expression lies a little upstream of promyelocytes [24,25]; these differentiating cells are otherwise almost inaccessible. This model is therefore well suited to the study of the response of neutrophil progenitor cells and mature neutrophils to various stimuli.

We here use this model to investigate the apoptotic response of neutrophil progenitors and mature neutrophils to roscovitine treatment. The results show a surprisingly strong role of the pro-apoptotic Bcl-2-family protein Noxa in progenitor cells and a strong role for the combination of the pro-apoptotic Bcl-2-family proteins Bim and Puma in differentiated cells. An improved understanding of stage-specific apoptosis-induction by roscovitine may facilitate the clinical use of this substance for the treatment of inflammatory disorders.

## Results

We established Hoxb8-dependent mouse neutrophil progenitor lines from a number of genetically modified mice. Cells used for this study had the genotypes wt, Bcl-2-transgenic (from vav-bcl-2-transgenic mice) or deficient for individual or combinations of pro-apoptotic BH3-only proteins (Bim^-/-^, Noxa^-/-^, Bim/Puma^-/-^ (double-deficient) and Bim/Noxa^-/-^ (double-deficient)). These proteins are the main candidates for roles in roscovitine-induced apoptosis and were selected on the basis of their role in neutrophil spontaneous apoptosis [24]. All of the cell lines were derived from C57Bl/6 mice, and most lines (all except the Bim^-/-^-line that was derived for this study) have been described previously [24].

**Figure 1:**
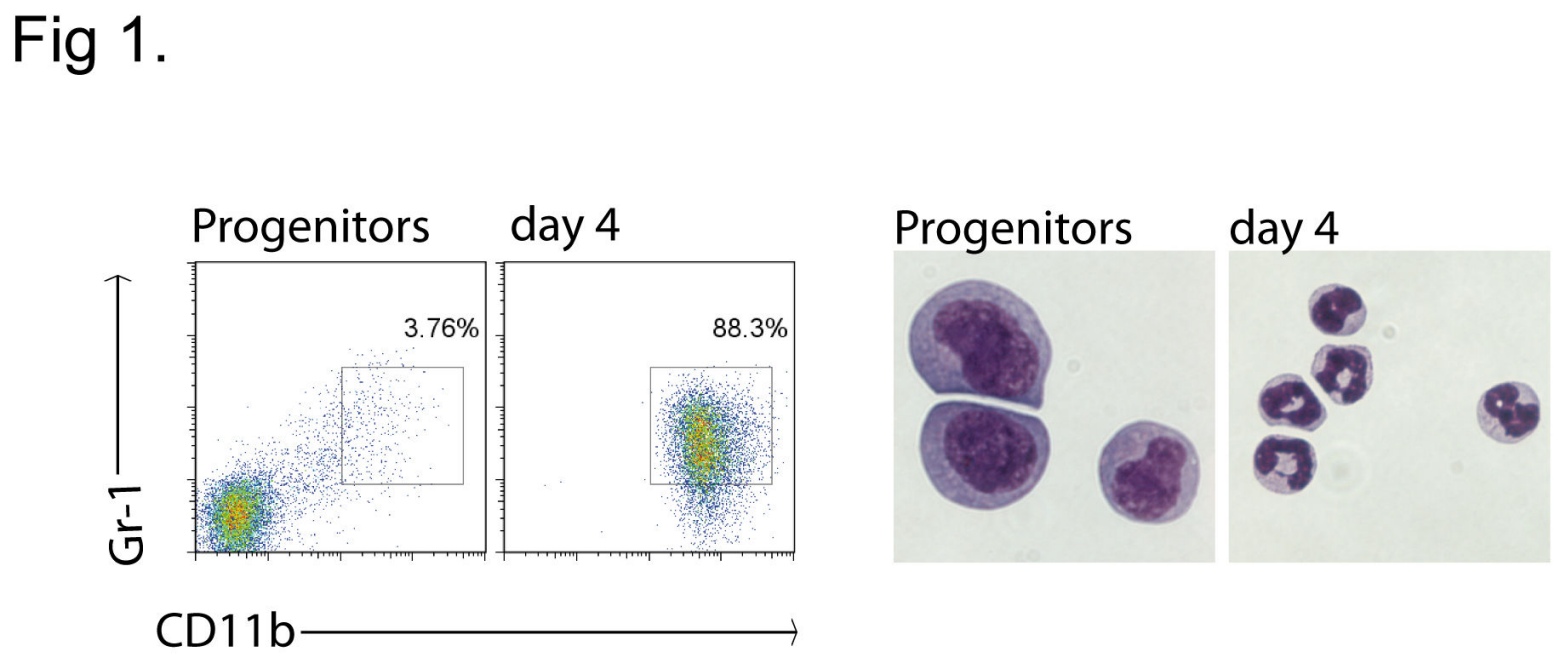
Example of the differentiation of Hoxb8 neutrophil progenitors. Left panel shows flow cytometer plots of Gr-1 and CD11b antibody staining of progenitors and day 4 differentiated wt neutrophils. Right panel shows Giemsa stain picture of wt progenitors and day 4 differentiated neutrophils, magnification 630X. Data are the representative of 3 independent experiments.

**Figure 2:**
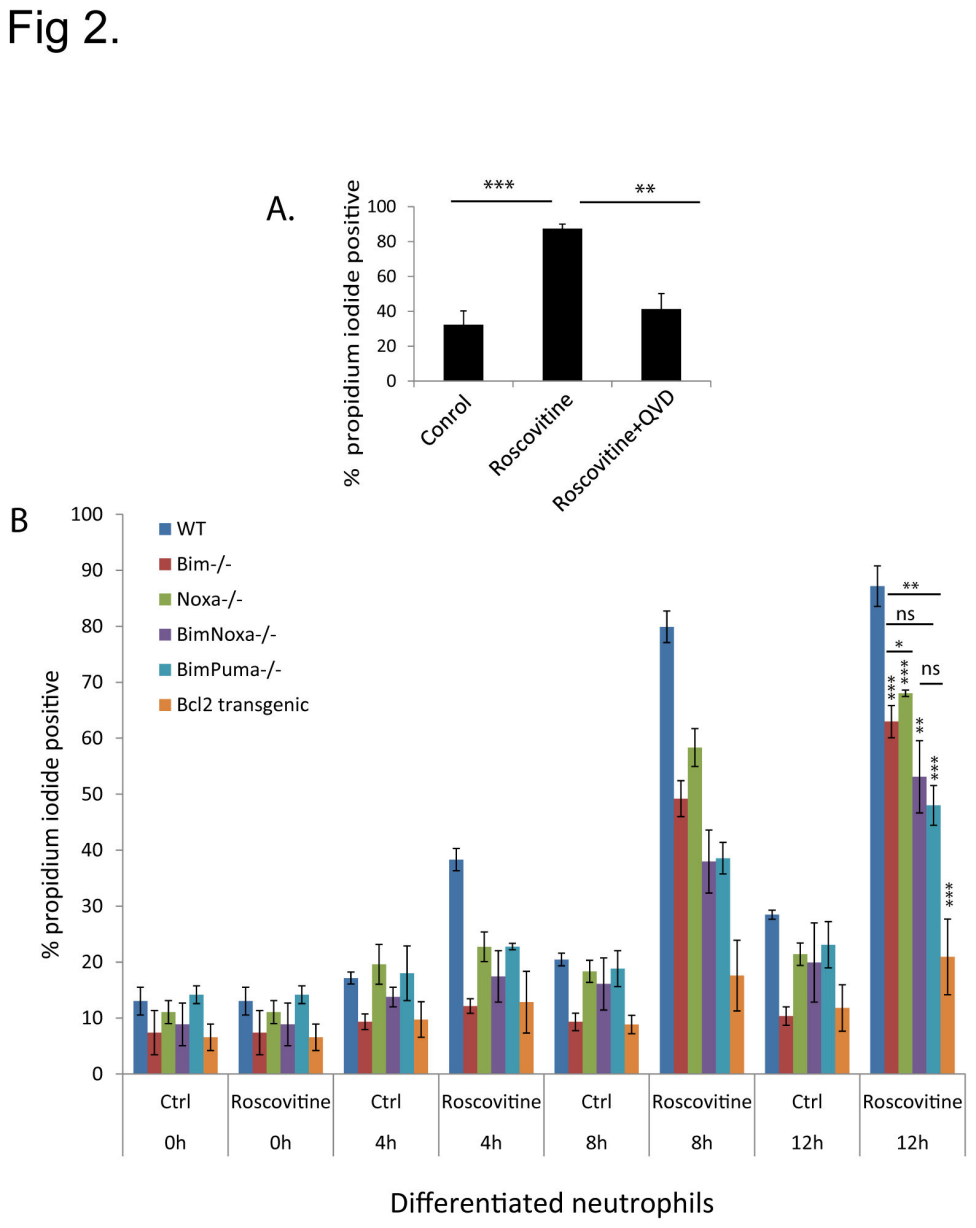
*Roscovitine induces* caspase dependent neutrophil apoptosis. (A) Propidium iodide (PI) staining of day 4 wt differentiated neutrophils treated with roscovitine (25 µM) alone or in the combination with the caspase-inhibitor Q-VD-OPh (50 µM) for 12h. (B) PI staining of wt, Bim^-/-^, Noxa^-/-^, Bim/Noxa^-/-^, Bim/Puma^-/-^, Bcl-2-transgenic differentiated neutrophils treated with Roscovitine (25 µM) for the times indicated. Bar diagrams represent means of 3 independent experiments and error bars indicate the standard error of mean (SEM). Statistical significance is calculated using Student’s t-test (ns, non-significant; *, p<0.05; **, p< 0.01; ***, p<0.001).

For the analysis of apoptosis-induction by roscovitine in mature neutrophils, the cells were differentiated by oestrogen-withdrawal for 4 days in the presence of SCF. An example of the expression of maturation markers (CD11b/Gr-1) and the morphology of progenitor and differentiated cells is given in Figure 1. Further maturation data of wt, Bcl-2-transgenic and Bim/Noxa-deficient lines is provided in [24]. As reported earlier, roscovitine induced massive apoptosis, and apoptosis was efficiently blocked by caspase-inhibition (Figure 2A). Cell death was inhibited by transgenic Bcl-2, confirming earlier results (Figure 2B) [6]. As a direct measure of apoptosis, we also analysed the activation of caspase-3 in wt and Bcl-2-transgenic neutrophils treated with roscovitine. There was clear activation of caspase-3 by treatment of roscovitine in wt but not in Bcl-2-transgenic mice (Figure S1).

The known involvement of the Bcl-2-family member Mcl-1 had suggested that roscovitine-induced apoptosis used the mitochondrial pathway, and the inhibition by Bcl-2 confirmed this. We therefore turned to a close examination of the members of the Bcl-2-family during roscovitine-induced apoptosis. In the immune system, Bim is the most important BH3-only protein (initiator of apoptosis) [18]. In lymphocytes Bim is to a degree redundant with Puma (the combined loss of Bim and Puma has much stronger effects than the loss of either protein alone) [26]. In NK cells and in neutrophils the loss of Noxa on its own has little effect but the combined loss of Bim and Noxa causes a strong resistance to spontaneous apoptosis [24,27].

To understand the involvement of BH3-only proteins in roscovitine-induced apoptosis we tested the susceptibility of neutrophils differentiated from Hoxb8-progenitors of various genotypes to cell death induced by this drug. As already said above, roscovitine-induced killing of wt cells was rapid, with about 85 % of wt cells dead after 12 h. This was of advantage to this study since the various genotypes show different levels of spontaneous apoptosis but spontaneous apoptosis is not high during this time period (from about 15 % at 0 h to 25-30 % at 12 h in wt cells, Figure 2B).

As shown in Figure 2B, the loss of Bim provided substantial but incomplete protection. The single loss of Noxa had a surprisingly strong effect, which was only slightly less pronounced than that of Bim-loss. The additional loss of either Puma or Noxa on a Bim-deficient background gave a relatively small but appreciable additional protection. The protection provided by Bcl-2 was superior to the one afforded by the loss of either combination of BH3-only proteins (Figure 2B). 

Mitochondrial apoptosis may be the result of increased levels of BH3-only proteins or the loss of anti-apoptotic proteins; strong changes in Bax or Bak are uncommon. To assess the main changes in Bcl-2-family proteins during roscovitine-induced apoptosis we used Bcl-2-transgenic cells. Since apoptosis is initiated in these cells but blocked by Bcl-2 before mitochondrial permeabilisation can occur, these cells show little apoptosis upon roscovitine-treatment and therefore the normal changes in Bcl-2-family members (such as BH3-only protein up-regulation, loss of anti-apoptotic proteins) can still be expected to occur normally in these cells, without the confounding effects of ongoing cell death.

**Figure 3:**
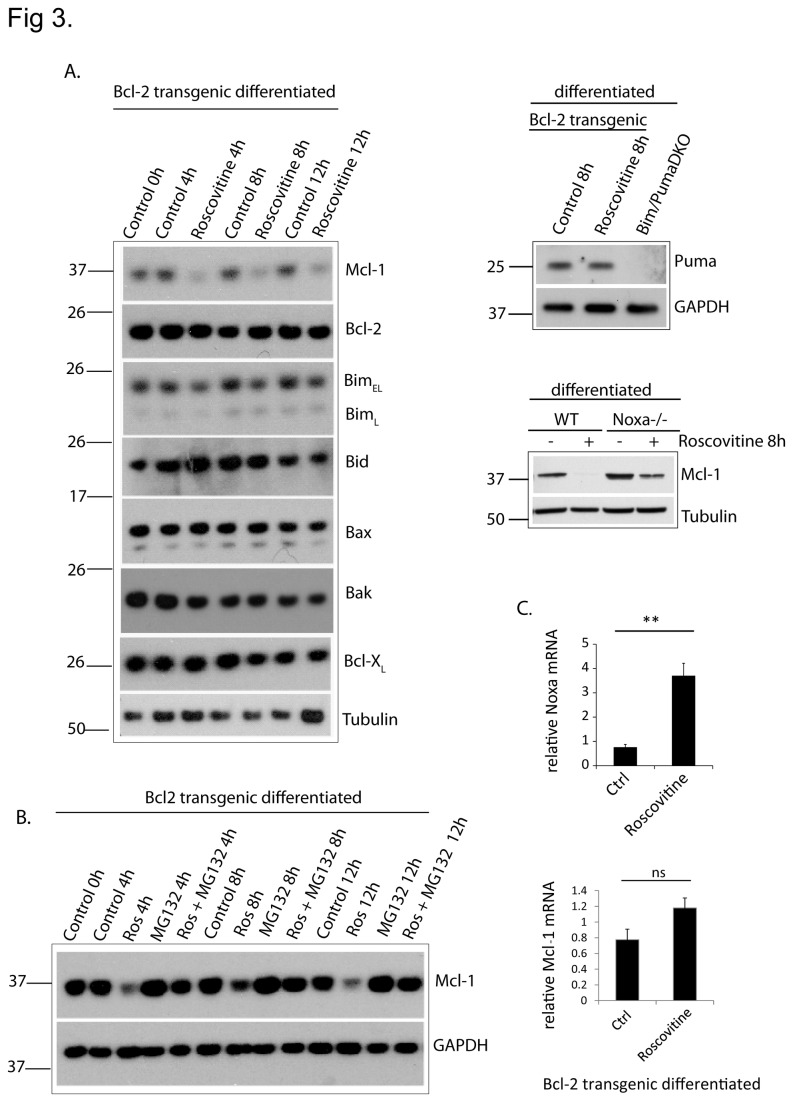
*Roscovitine causes* the loss of Mcl-1-protein but not -mRNA in differentiated neutrophils. (A) Western blot showing expression of Bcl-2 family proteins in day 4 differentiated Bcl-2-transgenic neutrophils after various times of roscovitine (25 µM) treatment. Mcl-1 expression is also shown in roscovitine-treated WT and Noxa-/- differentiated neutrophils.Tubulin or GAPDH serves as a loading control. Two experiments were performed with very similar results. (B) Western blot showing expression of Mcl-1 in day 4 differentiated Bcl2- transgenic neutrophils upon treatment for various times with roscovitine (Ros, 25 µM) and/or the proteasome-inhibitor MG-132 (0.2 µM). Expression of GAPDH was used as a loading control. The Western blot is representative of 2 independent experiments. (C) Graph shows noxa and mcl-1 transcript levels in day 4 differentiated Bcl-2- transgenic neutrophils treated with roscovitine for 4h. Data are mean/SEM of 3 independent experiments. Statistical significance was calculated using Student’s t-test (ns, non-significant; p > 0.05; **, p<0,01).

As expected, there were no substantial changes in Bax or Bak protein levels (Figure 3A). There were also no clear changes in the levels of Bcl-X_L_ or endogenous Bcl-2 (the transgene in *vav-bcl-2*-cells expresses human Bcl-2, which is not picked up by the anti-mouse Bcl-2-antibody we used) (Figure 3A). There was also no change in the levels of the BH3-only protein Bid and, despite its pro-apoptotic activity in this setting, we found no consistent up-regulation of Bim. Likewise, there was no obvious change in Puma protein levels (Figure 3A). There was, however, a striking loss of the anti-apoptotic protein Mcl-1 upon roscovitine-treatment (Figure 3A), similar to the loss reported in human neutrophils [15]. It therefore appears likely that, upon roscovitine-induced loss of Mcl-1, an inhibitory effect on Bim (and to a smaller degree on Puma) is released, which then permits the activation of Bax/Bak by Bim and subsequent apoptosis. 

In human neutrophils roscovitine has been found to down-regulate the Mcl-1 mRNA and to cause the loss of Mcl-1 by a mechanism that is blocked by the inhibition of the proteasome [12]. We found no down-regulation of Mcl-1-mRNA upon treatment of Bcl-2-expressing neutrophils with roscovitine (Figure 3C). However, proteasome inhibition almost completely maintained protein levels of Mcl-1 up to 12 h (Figure 3B). Roscovitine-treatment caused an about 4-fold up-regulation of Noxa mRNA-levels over 4 h (Figure 3C). Since one of the pathways causing Mcl-1-protein loss is through direct binding of Noxa [21] and consecutive proteosomal degradation, this suggests that at least part of the loss of Mcl-1 is due to Noxa-induction. Roscovitine-induced loss of Mcl-1-protein was reduced in Noxa-deficient cells, further arguing for this mechanism (Figure 3A).

**Figure 4:**
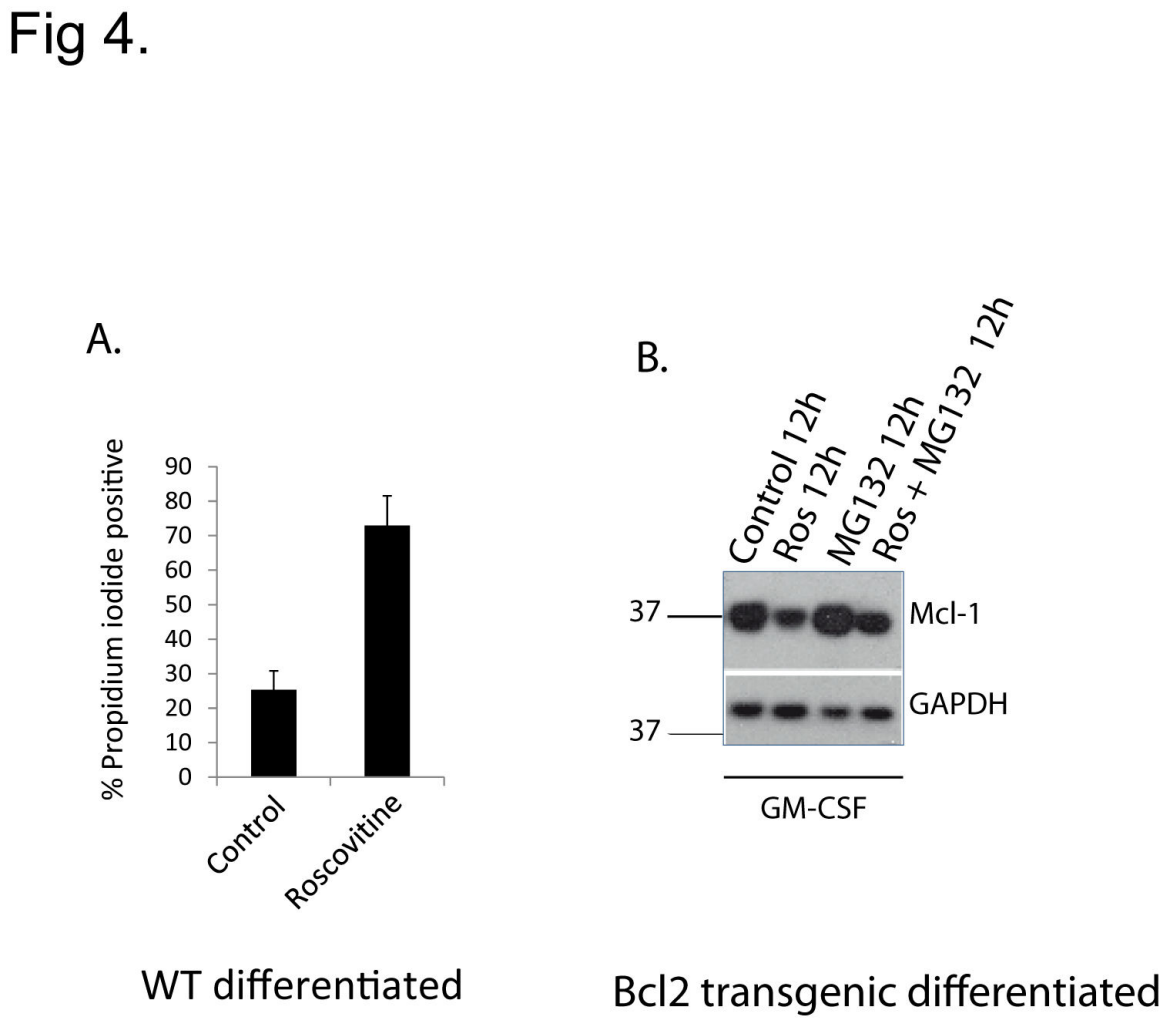
*Roscovitine overcomes* the pro-survival effect of GM-CSF. (A) PI-staining of day 4 differentiated wt neutrophils treated with roscovitine (25 µM) for 12 h in the presence of GM-CSF. Data are mean/SEM of 3 independent experiments. The data were generated in the same experiments as shown in Figure 2B and the control therefore is the same (B) Western blot showing Mcl-1 protein-levels in day 4 differentiated Bcl-2-transgenic neutrophils treated with roscovitine (Ros) (25 µM) and/or MG132 (0.2 µM) for 12 h in the presence of GM-CSF. Probing for GAPDH was used as a loading control. The Western blot is the representative of two independent experiments.

A number of pro-inflammatory cellular factors such as GM-CSF maintain Mcl-1-levels in neutrophils and can extend their lifespan. However, these agents could not protect human neutrophils against roscovitine-induced apoptosis [15]. We tested this link in our neutrophil model and found that GM-CSF had a modest protective effect when cells were treated with roscovitine (compare apoptosis-levels in Figures 4A and 2B). At the same time it had a modest effect in maintaining the levels of Mcl-1 (Figure 4). 

When roscovitine is considered as a therapeutic agent to induce apoptosis in neutrophils and thereby to reduce inflammation in the intact organism, another important consideration is the effect it may have on other cell types. Since neutrophils are exquisitely sensitive to roscovitine-induced apoptosis, and since the neutrophil population depends on constant supply from the bone marrow, the effect of systemic application of roscovitine on neutrophil progenitors may also be important. It is extremely difficult to investigate primary neutrophil progenitors; the population of myeloid progenitors in the bone marrow is a mixture of various, often small, subpopulations that cannot easily be purified [28]. The Hoxb8-system however offers the opportunity to study this population. Hoxb8-progenitors are committed neutrophil-progenitors that lie a little before promyelocytes and that, when differentiating into neutrophils, express the typical marker profile of myeloid cells differentiating along the neutrophil differentiation line (as far as this is known [23]). Although it is impossible at this stage to be sure that active Hoxb8 does not distort the expression pattern of apoptosis-relevant genes, there is no indication that it does. It appears at least arguable that these committed neutrophil progenitors behave like primary committed neutrophil progenitors. We therefore tested Hoxb8-progenitor cells for the requirements of Bcl-2-family members for roscovitine-induced apoptosis.

**Figure 5:**
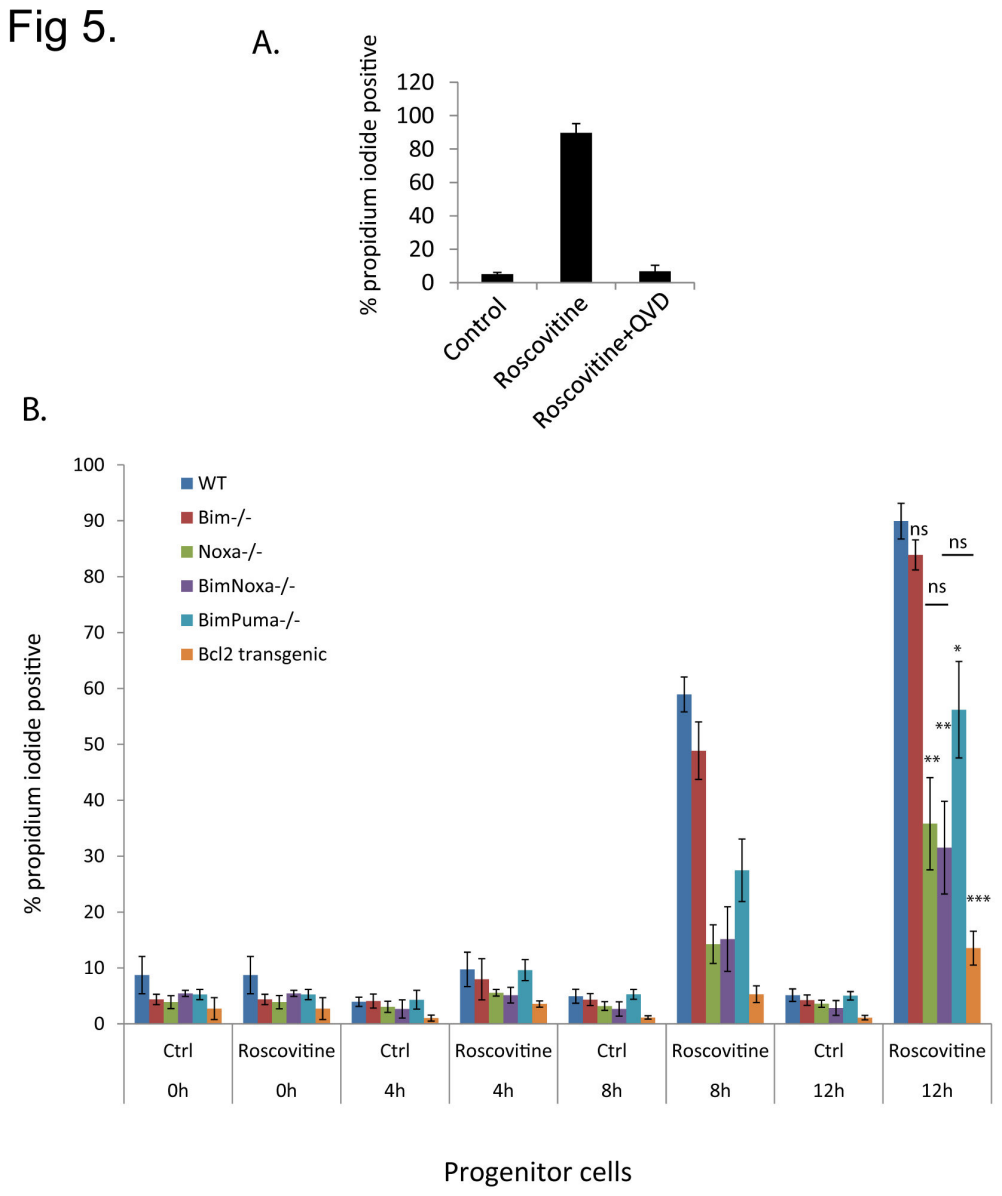
Role of caspases and BH3-only proteins in apoptosis in neutrophil progenitor cells. (A) PI-staining of wt progenitor cells treated with roscovitine (25 µM) alone or in the presence of the caspase-inhibitor Q-VD-OPh (50 µM) for 12 h. (B) PI staining of wt, Bim^-/-^, Noxa^-/-^, Bim/Noxa^-/-^, Bim/Puma^-/-^ and Bcl-2-transgenic progenitors treated with roscovitine (25 µM) for the indicated times. Bar diagram represents mean/SEM of three independent experiments. Statistical significance is calculated using the Student’s t-test (ns, non-significant; *, p<0.05; ** p< 0.01; *** p<0.001).

These cells were slightly less sensitive to roscovitine than differentiated cells, with about 60 % dying in the first 8 h but 90 % cell death at 12 h, which was inhibited by caspase-inhibition (Figure 5A, B). Bcl-2 again protected almost completely against roscovitine-induced apoptosis (Figure 5B). Surprisingly, the loss of Bim failed to protect these cells against roscovitine-induced apoptosis. When both Bim and Puma were absent, some protection was seen. A very strong effect was however seen when cells lacking Noxa were exposed to roscovitine, while additional loss of Bim did not further reduce sensitivity (Figure 5B).

**Figure 6:**
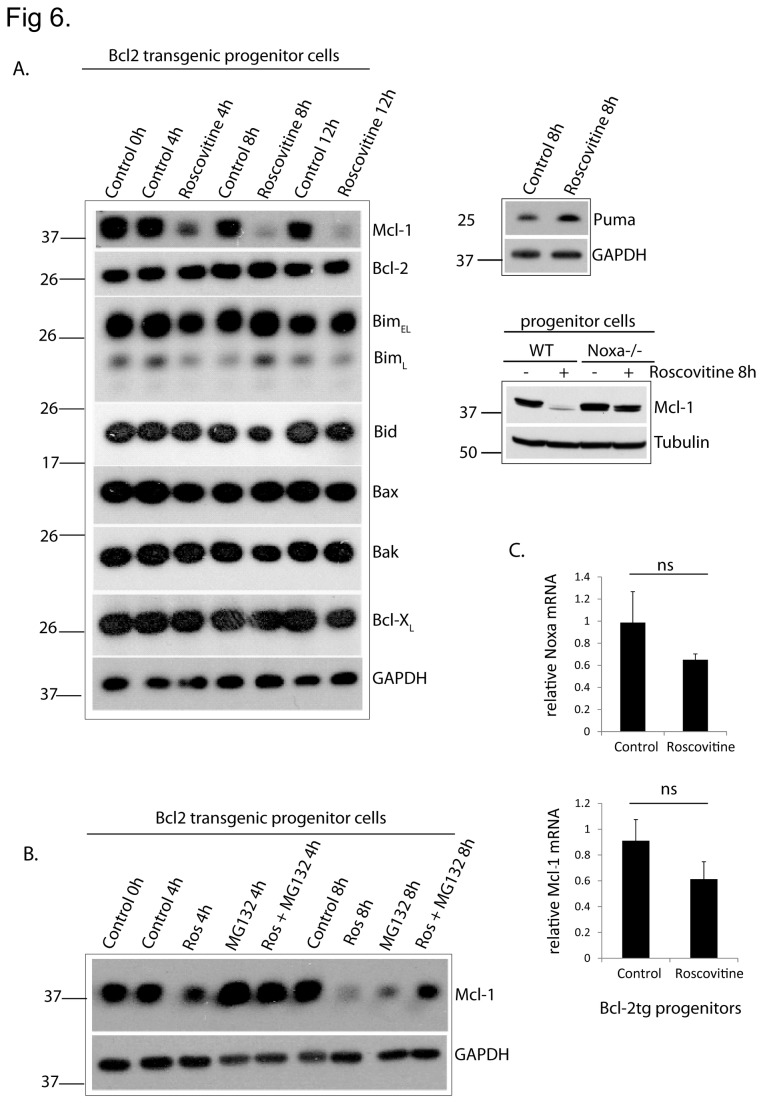
*Roscovitine causes* the loss of Mcl-1-protein in neutrophil progenitor cells. (A) Western blot showing expression of Bcl-2 family proteins in Bcl-2-transgenic progenitor cells at various times of roscovitine-treatment (25 µM). Mcl-1 expression is also shown in roscovitine-treated WT and Noxa-/- progenitors. Probing for GAPDH or tubulin served as a loading control. The experiment was performed twice with very similar results. (B) Western blot showing expression of Mcl-1 in Bcl2-transgenic progenitor cells at various times of roscovitine-treatment (25 µM) in the presence or absence of the proteasome-inhibitor MG132 (0.2 µM). Probing for GAPDH was done as a loading control. The blot is the representative of 2 independent experiments. (C) Graph shows noxa- and mcl-1-transcript levels in Bcl-2-transgenic progenitor cells treated with roscovitine for 4 h. Data are means/SEM of 3 independent experiments. Statistical significance was calculated using Student’s t-test (ns, non-significant).

As in mature neutrophils, the only Bcl-2-family protein whose expression showed a clear change was Mcl-1 (we again used Bcl-2-over-expressing cells to prevent cell death, Figure 6A). There was a small difference to mature cells in that the inhibition of Mcl-1-loss by proteasome-inhibition was less complete at 8 h (although equally strong at 4 h) but this seemed only a gradual effect. Neutrophil progenitors are thus also very sensitive to roscovitine-induced apoptosis. The molecular mechanisms are, based on the phenotypes of loss-of-function mutants, probably qualitatively similar but the quantitative contribution of the various pro-apoptotic proteins appears different. There was a small, non-significant reduction in Mcl-1 mRNA-levels but no change in Noxa mRNA-levels (Figure 6C; we were unable to detect Noxa-protein by Western blot). Unlike the situation in differentiated cells, roscovitine induced Puma-protein in progenitors (Figure 6A). This is consistent with the observed tendency that the difference between Bim/Puma-deficient and Bim-single-deficient cells was stronger in progenitors (where Puma was induced) than in differentiated cells (where Puma was not induced (compare Figures 2B and 5B for phenotype and 3A and 6A for protein levels). Although Noxa mRNA was not induced upon roscovitine-treatment, the loss of Mcl-1 was less severe in Noxa-deficient cells, probably accounting for the protection seen in these cells (Figure 6A). 

**Figure 7:**
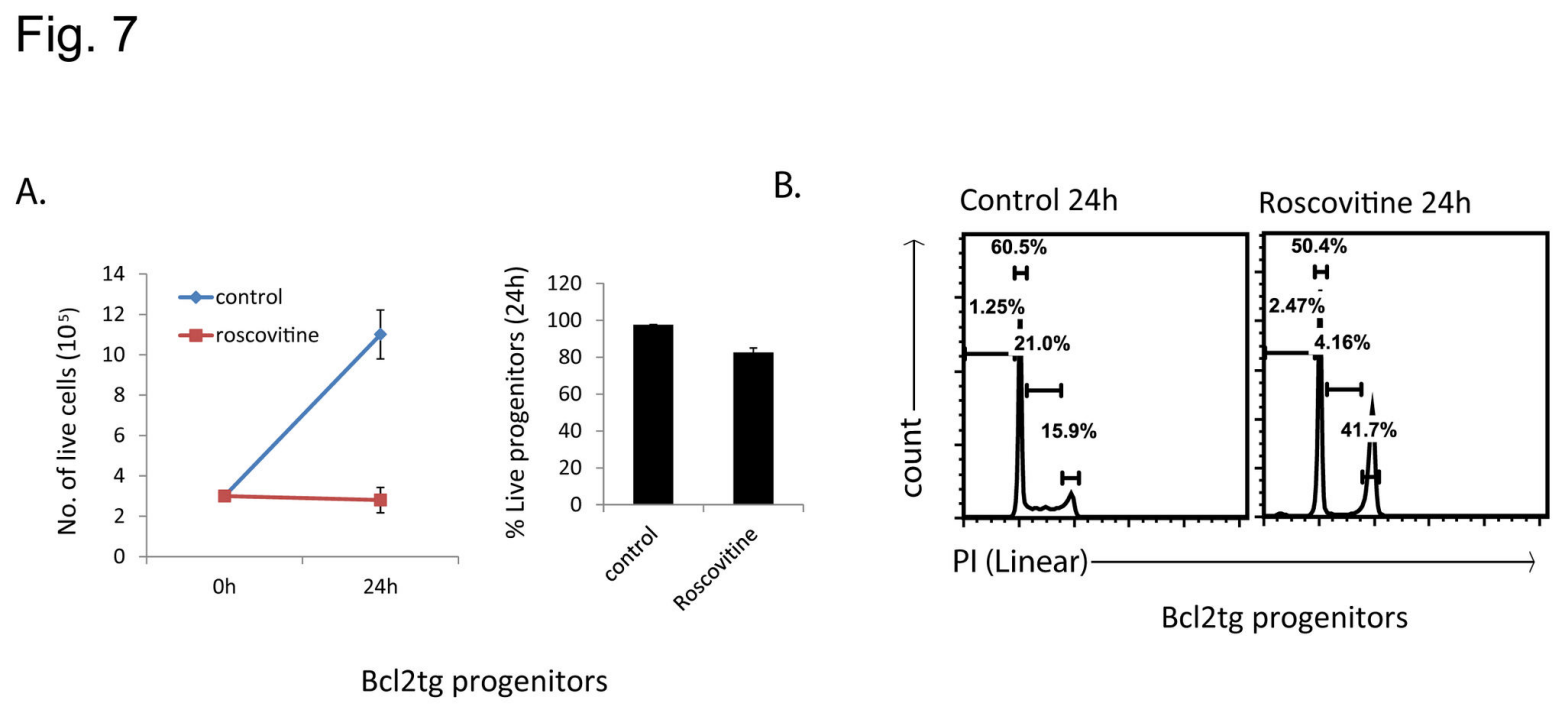
*Roscovitine inhibits* proliferation in neutrophil progenitor cells. (A) Left panel gives the numbers of Bcl-2-transgenic progenitor cells in control cultures and in cultures treated with roscovitine for 24 h. Right panel shows the percentages of live cells in these cultures (means/SEM of 3 independent experiments). (B) PI staining (flow cytometry analysis in linear scale) of Bcl-2-transgenic progenitor cells treated with roscovitine for 24 h. Flow cytometry plots are representative of 3 independent experiments.

Cyclin-dependent kinases are required for proliferation, and neutrophil progenitors proliferate rapidly. We tested whether the CDK-inhibitor roscovitine inhibited proliferation of neutrophil-progenitors. As shown in Figure 7A, Bcl-2-expressing neutrophil progenitors increased in number over 3-fold during 24 h of culture. Addition of roscovitine completely blocked this increase in population size. This was associated with substantial changes in cell cycle states: in the presence of roscovitine the population in S-phase was strongly reduced, accompanied by an increase in the G2/M-population. In neutrophil progenitors roscovitine thus causes both proliferation arrest and apoptosis, and this may have effects on the supply of mature neutrophils during apoptosis treatment *in vivo*.

## Discussion

We here demonstrate a number of molecular events and requirements of roscovitine-induced apoptosis in a mouse model of neutrophil-differentiation and show that, in this model, neutrophil progenitors also are sensitive to roscovitine, with similar requirements for Bcl-2-family members. A proliferation block in progenitors was also noted.

As mentioned above, the molecular events of mitochondrial apoptosis are still not completely clear. BH3-only proteins are the initiators of apoptosis, and the two main proteins involved in roscovitine-induced apoptosis in neutrophils, Bim and Noxa, probably have different molecular functions. Puma, which plays a smaller role, very likely has the same molecular activity as Bim.

Bim (and Puma) belongs to the group of ‘activator’ BH3-only proteins. These proteins can directly activate Bax, i. e. bind Bax through their BH3-domain and induce a conformational change that ultimately leads to the activation of Bax (oligomerisation and insertion into the mitochondrial outer membrane). This process is by now fairly well established [19]. The activation of Bak is less clear, and there is at this stage also the possibility that Bak is activated by ‘displacement’: when active BH3-only proteins displace anti-apoptotic proteins (in particular Mcl-1 and Bcl-X_L_) this has been proposed to be sufficient for the activation of Bak [21].

In all cases, it is likely that Bim (what is here said about Bim probably also applies to Puma) is activated and then activates Bax/Bak. Since the Bim-levels stay constant, this probably involves activation of already present Bim, and the most likely mechanism of Bim activation is the release from inhibition by anti-apoptotic Bcl-2-proteins. Since Mcl-1-levels drop rapidly in the presence of roscovitine, it is the most plausible scenario that Bim is normally bound to Mcl-1, released upon Mcl-1-degradation and then moves on to activate Bax/Bak.

If the loss of Mcl-1 is the triggering step, how is this achieved? Mcl-1-mRNA-levels did not drop and Mcl-1-protein-levels remained almost constant when proteasomal degradation was blocked, suggesting post-transcriptional regulation. This is at variance with the previously reported reduction of Mcl-1 mRNA-levels in human neutrophils during treatment with roscovitine [15]. This may be an inter-species difference. It is however also arguable that the observed apoptosis-associated loss in human neutrophils was a consequence of apoptosis and the loss of cellular integrity, rather than the cause of the drop in Mcl-1 protein levels. During apoptosis, cellular integrity is lost and numerous catabolic events take place, so it is difficult to separate initiating from secondary events. We used cells protected by Bcl-2, which did not undergo apoptosis but still down-regulated Mcl-1 protein without changes in Mcl-1 mRNA-levels. It therefore seems that loss of Mcl-1-protein, independent of transcription, is an early, very likely initiating, event of apoptosis. 

The results suggest a loss of Mcl-1 through post-transcriptional regulatory mechanisms. A number of mechanisms have been suggested in various cell-culture models. Post-translational modifications described include ubiquitylation through the ubiquitin-ligases FBW7 and Mule and de-ubiquitylation by the deubiquitinase Usp9x, as well as ubiquitination-independent degradation mechanisms [17,29]. Regulation through changes in translation has been little explored but remains a possibility.

Highly relevant in this context is the involvement of Noxa we observed. Noxa is a short BH3-only protein that inserts in the outer mitochondrial membrane and that has a high binding preference for Mcl-1 (and A1) [30,31]. Binding of Noxa to Mcl-1 causes its rapid proteasomal degradation. In differentiated cells this link appears straightforward as roscovitine-treatment induces Noxa mRNA, and the loss of Mcl-1 is smaller in Noxa-deficient cells. The regulation may be less obvious in progenitor cells, where the loss of Mcl-1 is also in part dependent on the presence of Noxa but there was no detectable induction of Noxa. Noxa in these cells is therefore required but the regulation of Mcl-1-levels probably also involves other mechanisms such as the ubiquitin-modifying enzymes described above.

The data suggest that a large part of roscovitine-induced neutrophil apoptosis is the result of Noxa-dependent degradation of Mcl-1, which results in the activation of Bim, which in turn activates Bax/Bak, culminating in cytochrome *c*-release and apoptosis. This leads us to propose a two-level model of roscovitine-induced apoptosis: first, Noxa-antagonism removes the protection by Mcl-1 through releasing Bim/Puma and secondly, the liberated Bim and Puma directly activate Bax.

Noxa plays only a small role in many situations of apoptosis investigated [32]. Indeed, the magnitude of the effect of Noxa-loss we observed in neutrophil progenitors is amongst the strongest instances reported to date. However, Noxa can specifically target Mcl-1, and since neutrophils probably from early stages very strongly depend on Mcl-1 [33], this strong effect of Noxa, especially in neutrophil progenitors, is perhaps not surprising. Despite the role of Noxa in reducing Mcl-1-levels upon roscovitine-treatment, we found no transcriptional up-regulation of Noxa. Recent work has shown that Noxa protein levels can be regulated by the proteasome in a lysine/ubiquitin-independent manner [34]. It may be such a post-translational mechanism that regulates Noxa-levels and -activity during roscovitine-treatment.

It has to be pointed out that these ‘progenitors’ still express active Hoxb8, and it is not impossible that this distorts the gene expression pattern. It is not entirely clear what Hoxb8 does to keep the cells immature. We have noticed that the neutrophil differentiation factor C/EBPα is expressed only at low levels in progenitor cells and goes up strongly during differentiation (unpublished). This enforces the validity of the cellular model, since a similar pattern is seen during differentiation of primary neutrophils [1]. Part of the function of Hoxb8 may therefore be the repression of C/EBPα.

Roscovitine also strongly inhibited proliferation of neutrophil progenitors. This proliferation-inhibition on the rapidly proliferating neutrophil progenitors may have additional effects *in vivo* on cells that have escaped apoptosis. Roscovitine may have a pro-apoptotic effect on neutrophil progenitors *in vivo* in the bone marrow, and this may affect neutrophil replenishment. It is possible that the bone marrow microenvironment protects the cells and mitigates apoptosis; in this case, the anti-proliferative effect may be more dominant *in vivo* but in either scenario roscovitine may affect the generation of mature neutrophils.

Our results thus illustrate some of the effects of apoptosis-induction by roscovitine, both in mature neutrophils and in neutrophil progenitors. The understanding of this process will help appreciate and further explore the potential of roscovitine (and other CDK-inhibitors) for the modulation of inflammation through the induction of neutrophil apoptosis. 

## Materials and Methods

### Cell lines and cell culture

Hoxb8 neutrophil progenitors were derived from bone marrow of WT, Bim^-/-^, Noxa^-/-^, Bim^-/-^/Puma^-/-^, Bim^-/-^/Noxa^-/-^ deficient and (human) *vav-bcl2*-transgenic mice on C57Bl/6 background as described earlier [24]. Cell lines were established by retroviral transduction of ER-Hoxb8 and selected in the presence of stem cell factor. Cell lines were polyclonal, and for these studies one polyclonal cell line per genotype was used in repeat experiments. 

Precursor cell lines were cultured in Optimem medium (Invitrogen) supplemented with 10% FCS Gold (PAA, Germany), 30 mM β-mercaptoethanol (Sigma), antibiotics (100 IU/ml penicillin G and 100 IU/ml streptomycin sulfate, PAA), 1% supernatant from stem cell factor (SCF)-producing Chinese Hamster Ovarian cells (gift from Dr. Hans Haecker, Memphis [23]), and 1 µM β-estradiol (Sigma). Neutrophil differentiation was initiated by removal of estrogen, followed by culture for 4 days in medium containing 1% SCF-supernatant. To assess the differentiation status, progenitors or day 4 differentiated neutrophils were washed in PBS and incubated in Fc block antibody (eBioscience) for 20 minutes prior to Gr-1 CD11b staining (anti-Gr-1-APC and anti-CD11b-PE antibodies were from eBioscience) for 20 min on ice. Stained neutrophils were then analyzed by flow cytometry. Giemsa stain (Sigma Aldrich) was performed on methanol fixed wt progenitors or day 4 differentiated neutrophils for 40 minutes followed by tap water wash. 

### Apoptosis assays

For cell death experiments, cells were treated with 25 µM roscovitine in the presence or absence of 50 µM pan-caspase inhibitor qVD-OPH (R&D Systems) or 0.2 µM MG-132 (Enzo Life Sciences) or treated with solvent control. Cell death was assessed by propidium iodide (PI) staining (1 µg/ml) followed by flow cytometer analysis. In some cultures GM-CSF was added (1 % culture supernatant from a stably transfected, GM-CSF-producing B16 cell line [approximately 10 ng/ml] as described [23]).

For active caspase-3 staining, control and roscovitine treated neutrophils were washed, fixed in 4% formaldehyde and permeabilized with 0.5% Saponin (Sigma, Munich, Germany) in PBS. Antibody staining was performed with anti-active caspase-3 (BD Pharmingen, Heidelberg, Germany) in PBS/0.5% BSA for 20 min at room temperature, followed by incubation with anti-rabbit-Cy5 (Dianova, Hamburg, Germany) for 20 min. After washing the pellets, samples were analyzed by flow cytometry.

### RNA extraction and quantification

Total RNA was extracted and transcribed into cDNA with commercial kits (Roche) following the manufacturer’s instructions. Samples were analyzed by quantitative RT-PCR using the Light Cycler TaqMan Master Kit and the Universal Probe Library system (Roche). Relative expression of the gene of interest (Mcl-1 or Noxa) was normalized to β-actin reference gene expression.

### Immunoblot analysis

Cells were harvested and immediately lysed in Laemmli buffer (3 x 10^6^/100 µl). After separation of cell extracts by SDS-PAGE, proteins were transferred onto PVDF membranes. Loading of equal cell numbers was confirmed by probing for GAPDH or tubulin using specific antibodies (Millipore, Sigma). Membranes were probed with antibodies against mouse Bim, Bcl-X_L_, Bax, Puma (all from Cell Signaling Technology), Bid, (NT, ProSci), Bak (Millipore), Bcl-2 (BD Pharmingen) or Mcl-1 (Rockland; Epitomics). Proteins were visualized using peroxidase-conjugated secondary antibodies against rabbit (Sigma), mouse, rat or hamster (all from Dianova) and an enhanced chemoluminescence detection system (GE Healthcare).

### Cell cycle assay

Roscovitine treated/untreated 24h Bcl2 transgenic Hoxb8 neutrophil progenitors were fixed in 80% ice cold methanol for 1 h followed by RNAse (0.5 mg/ml) treatment for 1 h at 37° C. Samples were then incubated in propidium iodide and analyzed by flow cytometry.

## Supporting Information

Figure S1 **Activation of caspase-3 by roscovitine-treatment.** Wt or Bcl-2-transgenic differentiated neutrophils were cultured in the absence or presence of roscovitine for 8 h. Active caspase-3 was detected by intracellular staining with specific antibody. The left panels show original flow cytometry blots from one experiment, the right one gives mean/SEM of three independent experiments. Statistical significance is calculated using Student’s t-test (**, p <0,01).
(TIF)
