# Correction: Roscovitine-induced Apoptosis in Neutrophils and Neutrophil Progenitors Is Regulated by the Bcl-2-Family Members Bim, Puma, Noxa and Mcl-1

**DOI:** 10.1371/annotation/5188aea6-9eb2-41e5-995b-86634bf3e0cf

**Published:** 2014-01-03

**Authors:** Sanjivan Gautam, Susanne Kirschnek, Michael Wiesmeier, Juliane Vier, Georg Häcker

Two affiliations of the first author were incorrectly omitted from the byline. Sanjivan Gautam affiliated not only with institution number 1, but with 2, Spemann Graduate School of Biology and Medicine, University of Freiburg, Germany, and 3, Faculty of Biology, University of Freiburg, Germany. 

