# Supplementary figures and images for: Roscovitine-induced Apoptosis in Neutrophils and Neutrophil Progenitors Is Regulated by the Bcl-2-Family Members Bim, Puma, Noxa and Mcl-1
Source: PLoS One. 2013 Nov 1;8(11):e79352. doi: 10.1371/journal.pone.0079352 (PMC3815126; doi:10.1371/journal.pone.0079352)

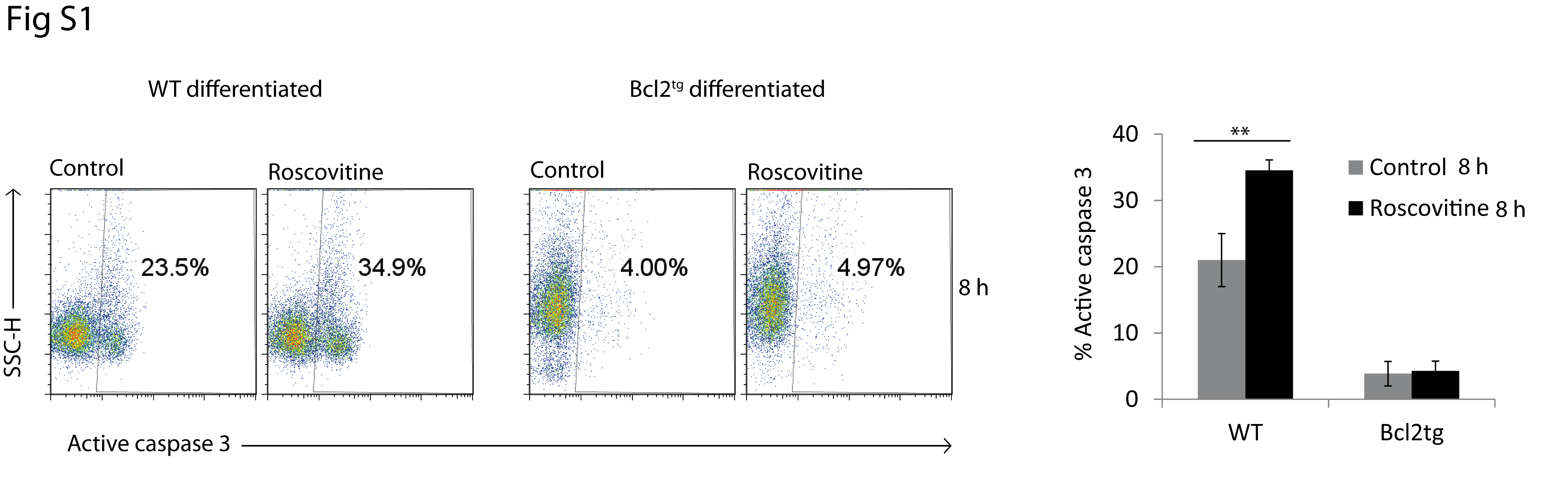

Supplement: Figure S1 — Activation of caspase-3 by roscovitine-treatment. Wt or Bcl-2-transgenic differentiated neutrophils were cultured in the absence or presence of roscovitine for 8 h. Active caspase-3 was detected by intracellular staining with specific antibody. The left panels show original flow cytometry blots from one experiment, the right one gives mean/SEM of three independent experiments. Statistical significance is calculated using Student’s t-test (**, p <0,01). (TIF) [file pone.0079352.s001.tif]
